# Prognostic Implication of a CT-FFR-Based Functional SYNTAX Score in Coronary Artery Disease Patients Undergoing Percutaneous Coronary Intervention

**DOI:** 10.3390/jcm15207826

**Published:** 2026-10-10

**Authors:** Qiaoyu Shao, Uwe Joseph Schoepf, Xiaoteng Ma, Lei Xu, Yujie Zhou, Fei Gao, Zhijian Wang

**Affiliations:** 1 Department of Cardiology, Beijing Anzhen Hospital, Capital Medical University, Beijing 100029, China; shaoqy@163.com (Q.S.);; 2 Division of Cardiovascular Imaging, Medical University of South Carolina, Charleston, SC 29425, USA; 3 Department of Radiology, Beijing Anzhen Hospital, Capital Medical University, Beijing 100029, China

**Keywords:** functional SYNTAX score, percutaneous coronary intervention, risk stratification

## Abstract

**Background**: This study aims to investigate the value of a SYNTAX score (SS) based on coronary computed tomographic angiography (cCTA)-derived fractional flow reserve (CT-FFR), termed “Functional SYNTAX score” (FSS_CTA_), to predict major adverse cardiovascular events (MACEs) in patients undergoing percutaneous coronary intervention (PCI). **Methods**: This cohort study included 876 patients who underwent cCTA and underwent PCI within 30 days during 2016–2020. CT-FFR was calculated by a machine learning algorithm. All 876 patients were divided into a low-risk group (≤22) and an intermediate-to-high-risk group (>22) with cCTA-based SS (SS_CTA_) as the control. **Results:** Based on the FSS_CTA_, 10.4% (91/876) of patients were reclassified to the low-risk group, and 2.9% (25/876) of patients were reclassified to the intermediate-to-high-risk group. MACEs occurred in 16.6% (145/876) of patients over a median follow-up period of 19.4 months. FSS_CTA_ and invasive coronary angiography-based SS (SS_ICA_) were independently associated with the risk of MACEs regardless of whether they were in continuous or categorical form (FSS_CTA_: c continuous HR 1.082, 95% CI 1.038–1.128; categorical HR 1.865, 95% CI 1.138–2.305. SS_ICA_: continuous HR 1.045, 95% CI 1.012–1.079; categorical HR 1.520, 95% CI 1.128–2.048). FSS_CTA_ showed the best predictive power for MACEs compared with SS_CTA_ and SS_ICA_ (all comparisons with FSS_CTA_ *p* < 0.05). **Conclusions**: FSS_CTA_, SS_CTA_ and SS_ICA_ were all independent predictors of MACEs in patients undergoing PCI, and FSS_CTA_ showed a significantly better predictive value for MACEs than SS_CTA_ and SS_ICA_.

## 1. Introduction

The SYNTAX score (SS) is a scoring system derived from the SYNTAX study (SYNergy between percutaneous coronary intervention with TAXus and cardiac surgery) [1]. The SS quantitatively evaluates the complexity of coronary artery stenosis considering the lesion localization, severity, bifurcation pattern, calcification, and severe tortuosity. Scores of 22 and 32 are commonly used thresholds for low, intermediate, and high anatomical complexity. The SS was recommended by American and European revascularization guidelines as a basis for making revascularization strategies due to its capability to predict the postprocedural risk of percutaneous coronary intervention (PCI) or coronary artery bypass grafting (CABG) [2,3]. The functional SYNTAX score (FSS) is a modification of SS incorporating the functional measurement of fractional flow reserve (FFR). In the Fractional Flow Reserve versus Angiography for Multivessel Evaluation (FAME) study [4], recalculating the SS by solely calculating ischemic-specific lesions as determined by FFR would move 34% of intermediate-to-high-risk patients to lower-risk groups and better predict the risk of major adverse cardiovascular events (MACEs) for patients with multivessel coronary artery disease (CAD) undergoing PCI. However, performing FSS measurements manually on all lesions is not feasible in clinical practice considering that conventional SS is a complex and subjective process and FFR is an invasive and costly assessment. Therefore, there is an urgent need for a non-invasive and efficient functional assessment method to assist in the diagnosis and management of CAD.

Coronary computed tomographic angiography-derived fractional flow reserve (CT-FFR) values can be calculated for any point of the entire coronary tree without additional computed tomographic (CT) studies and pharmacological stress. The diagnostic accuracy of CT-FFR for identifying functionally relevant coronary stenosis lesions has been demonstrated in several multicenter studies [5,6,7], even in heavily calcified lesions [8]. CT-FFR was also shown to correlate well with invasive FFR measured during invasive coronary angiography (ICA) or PCI [9,10]. However, it is still worth investigating the predictive value of CT-FFR-based FSS (FSS_CTA_) by combining SS with CT-FFR on the clinical prognosis of CAD patients undergoing PCI. This study sets out to assess the predictive value of FSS_CTA_ calculated by artificial intelligence (AI)-based CT-FFR analysis for MACEs in patients with CAD undergoing PCI.

## 2. Materials and Methods

### 2.1. Study Design

**Figure 1:**
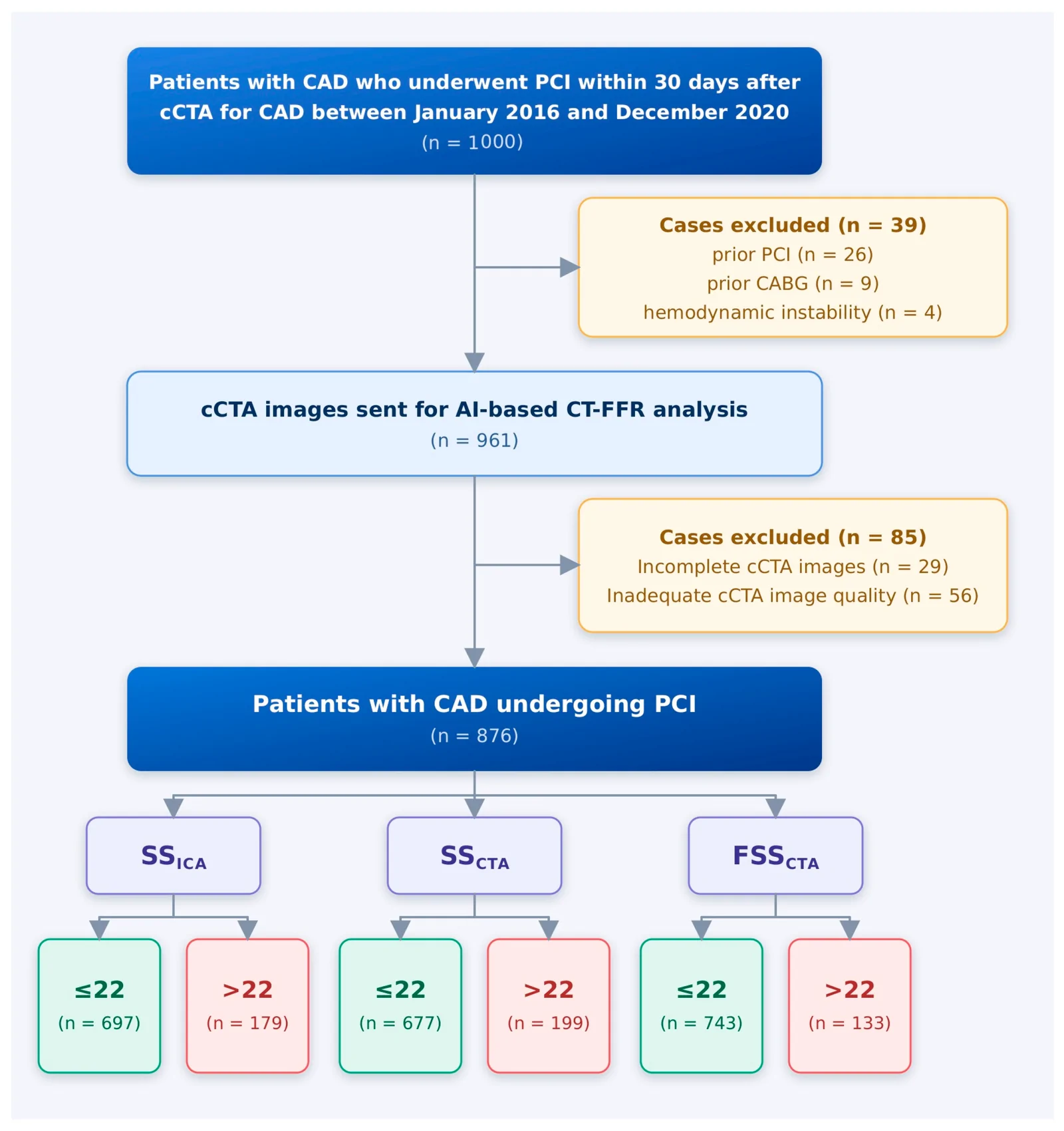
Flow chart of patient enrollment, exclusion, and classification. Abbreviations: AI, artificial intelligence; CABG, coronary artery bypass grafting; CAD, coronary artery disease; cCTA, coronary computed tomographic angiography; CT-FFR, coronary computed tomographic angiography-derived fractional flow reserve; FSS_CTA_, functional SYNTAX score based on CT-FFR; PCI, percutaneous coronary intervention; SS_CTA_, coronary computed tomographic angiography-based SYNTAX score; SS_ICA_, invasive coronary angiography-based SYNTAX score.

This retrospective cohort study recruited 1000 consecutive patients with CAD who underwent PCI within 30 days after coronary computed tomographic angiography (cCTA) between January 2016 and December 2020. This study was approved by the Institutional Review Committee of Beijing Anzhen Hospital, Capital Medical University, and complied with the “Helsinki Declaration of Human Rights”. Inclusion criteria were ≥50% stenosis in at least one coronary artery with diameter ≥ 1.5 mm on cCTA. Exclusion criteria included history of prior PCI, history of CABG; hemodynamic instability; incomplete cCTA images; and inadequate cCTA image quality. We ultimately enrolled 876 patients with CAD undergoing PCI whose images were successfully analyzed by AI-based CT-FFR (Figure 1). This study was designed as a retrospective cohort study with consecutive enrollment, and the sample size was therefore determined by the number of consecutive eligible patients available during the study period rather than by a prospective sample size calculation. A post hoc power analysis based on the observed effect size for the categorical FSS_CTA_ (>22 vs. ≤22) in the multivariable Cox model (adjusted HR 1.865) indicated that approximately 81 events were required for 80% power at a two-sided α of 0.05. The present study observed 145 primary MACEs, which exceeds this requirement, and the EPV ratio was approximately 14.5, satisfying the conventional EPV ≥ 10 criterion.

### 2.2. Data Collection

All cCTA studies were acquired using a 256-row CT scanner (Revolution CT, GE Healthcare, Milwaukee, WI, USA) or a dual-source CT scanner (Somatom Definition Flash, Siemens, Forchheim, Germany). The cCTA-based diagnosis was determined by two senior cardiovascular imaging specialists who were blinded to clinical information. Within 30 days, ICA was performed by a group of experienced interventional cardiologists following the standard protocol to obtain at least two different views for each major vessel so as to determine the degree of coronary stenosis. Treatment decisions including PCI, CABG, and/or optimal medical therapy were determined during the ICA procedure independently without the information from this study. The degree of stenosis based on cCTA or ICA was classified into 3 grades: mild (0–49%); moderate (50–69%); and severe (70–99%).

The CT-FFR analysis was independently performed blinded to patients’ clinical information as well as ICA results using DEEPVESSEL FFR software (version 1.0, Keya Medical, Shenzhen, China). This software used deep learning technology to evaluate non-invasive FFR from coronary CT angiography images. The software firstly utilized deep learning approaches to segment and extract the aorta and coronary arteries [11]. User interaction might be needed to remove connected veins if necessary. The centerlines of the coronary arteries were then extracted to represent the coronary topology structure [12]. Lastly, a tree structured deep learning model directly calculated CT-FFR from the embedded anatomical and imaging information from the complex coronary artery tree for each centerline point [13].

SS calculations were performed separately for all patients according to ICA, cCTA, and CT-FFR by an online SS calculation software (SYNTAX Score Calculator, version 2.28, available at http://www.syntaxscore.com, accessed between November 2023 to August 2024). The complexity of coronary stenosis was assessed quantitatively according to the SS rule for all lesions with ≥50% stenosis on vessels ≥ 1.5 mm in diameter [1]. The ICA-based SS (SS_ICA_), cCTA-based SS (SS_CTA_), and FSS_CTA_ were calculated and used to classify 876 patients into low-risk (≤22) and intermediate-to-high-risk (>22) groups, respectively [4]. The SS_ICA_ was scored by experienced interventional cardiologists after interpreting the angiographic films. The SS_CTA_ and FSS_CTA_ were calculated by cardiovascular radiologists and the data analysis team from the DEEPVESSEL FFR platform. All three scores were calculated by at least two independent experts, and a third senior expert was involved when discrepancies occurred.

Each coronary lesion with ≥50% stenosis in a vessel ≥ 1.5 mm in diameter was identified on cCTA and assigned an anatomical SYNTAX score according to the standard SYNTAX algorithm, and the FSS_CTA_ was calculated as follows. CT-FFR was computed for the entire coronary tree using the DEEPVESSEL FFR software (Keya Medical, China). For each anatomically significant lesion, the minimum CT-FFR value within the lesion segment (from the proximal to the distal end of the lesion, as defined on cCTA) was used to determine functional significance. A lesion was considered functionally significant if the minimum CT-FFR within the lesion was ≤0.80. Lesions with a minimum CT-FFR > 0.80 were assigned zero points in the FSS_CTA_ calculation, even if anatomically significant. The FSS_CTA_ was then calculated as the sum of anatomical SYNTAX points for functionally significant lesions only. For serial lesions within the same vessel, each lesion was assessed separately using its own minimum CT-FFR value. For bifurcation lesions, the standard SYNTAX bifurcation rules were applied. Vessels with a diameter < 1.5 mm were not scored, consistent with the standard SYNTAX methodology.

To ensure reader blinding and reproducibility, CT-FFR analysis was performed by an independent analysis team (Keya Medical) blinded to all clinical information, ICA results, and patient outcomes. SS_CTA_ and FSS_CTA_ were calculated by cardiovascular radiologists and the data analysis team, also blinded to clinical outcomes. SS_ICA_ was scored by two experienced interventional cardiologists blinded to the CT-FFR and FSS_CTA_ results. For all 876 patients, SS_ICA_, SS_CTA_, and FSS_CTA_ were independently scored by two experts. When the two experts disagreed, a third senior expert was involved, and the final consensus score was used in the analysis. To assess interobserver reproducibility, the intraclass correlation coefficient (ICC) was calculated by comparing the original independent duplicate readings of the two readers for each score, before adjudication. The ICC was 0.87 for SS_ICA_, 0.81 for SS_CTA_, and 0.83 for FSS_CTA_, indicating good to excellent interobserver agreement.

Patient information was obtained by reviewing medical records, and the follow-up data were collected by telephone contact. Patients were consecutively enrolled between January 2016 and December 2020. The median follow-up was 19.4 months (IQR 12.5–25.6). The variation in follow-up duration reflects differences in the timing of enrollment, with earlier enrollees having longer follow-up. The loss-to-follow-up rate was 0%. The demographics and risk factor data were retrospectively recorded on admission. Fasting total cholesterol > 5.17 mmol/L, low-density lipoprotein-cholesterol > 3.36 mmol/L, triglycerides > 1.69 mmol/L, high-density lipoprotein-cholesterol < 1.03 mmol/L, and/or chronic use of lipid-lowering drugs were considered criteria for dyslipidemia. Blood samples were collected after 12 h of fasting during hospitalization to obtain high-sensitivity C-reactive protein, lipid profile, fasting plasma glucose, glycosylated hemoglobin, and creatinine levels. The Global Registry of Acute Coronary Events (GRACE) score was calculated on admission for predicting 6-month death or MI. Medication data were collected from primary drugs taken by patients at discharge.

### 2.3. Endpoint Definitions

All endpoints were independently adjudicated by a blinded event committee. The primary endpoint of the present study was a composite of MACEs, including all-cause mortality, non-fatal myocardial infarction (MI), non-fatal ischemic stroke, or unplanned repeat revascularization. MI was defined as elevated cardiac troponin or creatine kinase levels with ischemic symptoms or ischemic electrocardiograph changes, including new pathological Q waves in ≥2 consecutive leads. Non-fatal stroke was defined as an ischemic cerebral infarction with neurological deficits requiring hospitalization and a clinically documented lesion on computed tomography or magnetic resonance imaging of the brain. Unplanned repeat revascularization was defined as any non-staged revascularization beyond 90 days after index PCI, or if the revascularization was urgent, emergency, or salvage. Planned staged PCI (planned at the time of index PCI and performed during the index hospitalization or within 90 days) was not counted as unplanned repeat revascularization. The definition of repeat revascularization encompassed target-lesion, target-vessel, and non-target-vessel revascularization. When more than one component occurred during follow-up, only the most severe component (death > stroke > MI > revascularization) was reported in the descriptive analysis of the composite endpoint in order to avoid double-counting the same patient. For all time-to-event analyses, however, the event time was defined as the interval from the index PCI to the first occurrence of any qualifying MACE component. Patients who did not experience any component were censored at the date of last follow-up.

### 2.4. Statistical Analysis

The normality of continuous variables was assessed by Quantile–Quantile plots. Continuous variables in normal distributions were presented as means ± standard deviations by an independent-sample *t*-test, and variables in non-normal distributions were presented as medians and interquartile ranges (IQR) by the Mann-Whitney U test. Categorical variables were expressed as counts and percentages. The comparison between groups was examined by a Chi-squared test or Fisher’s exact test (categorical variables) and ANOVA or the Kruskal–Wallis H test (continuous variables). The Kaplan–Meier method was used to plot the time–survival curve. For all time-to-event analyses, the event time was defined as the time to the first occurrence of any composite MACE component. The unadjusted and adjusted Cox proportional hazards models were used to assess the association among the FSS_CTA_, SS_CTA_, and SS_ICA_ (considered as a continuous variable and categorical variable) and MACEs. The proportional hazards assumption was assessed using Schoenfeld residuals, and no significant violation was observed. The linear relationship between each continuous SYNTAX score and log hazard was assessed using restricted cubic splines, with no evidence of non-linearity. In the hard MACE sensitivity analysis, the multivariable model was adjusted for age and sex (Supplementary Table S1). Covariates were prespecified based on clinical relevance and established prognostic importance in CAD patients undergoing PCI, rather than solely on univariable *p*-value screening. To avoid collinearity, highly correlated variables were not included simultaneously: diabetes was retained as the clinical variable (fasting glucose, HbA1c, and insulin use were not included), and chronic kidney disease (CKD) was retained as the renal variable (creatinine was not included). Variance inflation factors were all <5. A more parsimonious sensitivity model (age, sex, GRACE score, and complete revascularization only) yielded consistent results. The number of covariates was limited to approximately 10, consistent with the events-per-variable criterion. Each SYNTAX score was evaluated in a separate adjusted model (SS_ICA_ + baseline model, SS_CTA_ + baseline model, and FSS_CTA_ + baseline model) because the three scores are highly correlated. Complete revascularization and discharge medications were not included in the primary model, as they are post-baseline variables that may act as mediators rather than confounders; a secondary postprocedural model incorporating these variables was also performed (Supplementary Table S2). The results of survival analyses were presented as the hazard ratio (HR) and 95% confidence interval (CI). The incremental predictive values were analyzed by calculating C-statistics, net reclassification improvement (NRI) and integrated discrimination improvement (IDI). The baseline model used for the incremental predictive-performance analysis was the same preprocedural model as the primary analysis (age, sex, BMI, current smoking, hypertension, diabetes, dyslipidemia, CKD, and GRACE score), defined a priori to ensure consistency and to avoid including post-baseline variables that may act as mediators. Discrimination was assessed using Harrell’s C-statistic, and comparisons were performed using an established method for correlated C-statistics. The time horizon for NRI and IDI was set at 18 months. NRI was calculated as a continuous NRI, and IDI was calculated using survival-based methods with inverse probability of censoring weighting. Confidence intervals for NRI and IDI were derived using bootstrap resampling (1000 replicates). Internal validation was performed using bootstrap resampling to obtain optimism-corrected C-statistics. Calibration was assessed using calibration plots, calibration slope, and the Brier score (Supplementary Table S3). The 2-sided significance level was set at *p* < 0.05. All statistical analyses were performed with R Software version 4.1.0 (R Foundation for Statistical Computing, Vienna, Austria) and IBM SPSS Statistics version 26.0 (IBM Corporation, Chicago, IL, USA).

## 3. Results

### 3.1. Baseline Characteristics

The mean age of all 876 patients was 62 ± 10 years, and 73.1% were male. One-vessel disease was present in 312 patients (35.6%), two-vessel disease in 298 (34.0%), three-vessel disease in 266 (30.4%), and left main disease in 98 (11.2%). Clinical presentation was stable CAD in 412 patients (47.0%), unstable angina in 341 (38.9%), and non-ST-elevation myocardial infarction (NSTEMI) in 123 (14.0%). The median interval between cCTA and PCI was 8 days (IQR 4–15). Follow-up data were collected for all patients with a median follow-up period of 19.4 (IQR 12.5–25.6) months. Whether SS greater than 22 was based on ICA, CTA, or CT-FFR; patients had higher GRACE scores, total cholesterol, and fasting glucose levels and a lower percentage of complete revascularization. When the anatomical SS was greater than 22, patients had higher glycosylated hemoglobin levels and a higher proportion of β-blockers use. Patients with FSS_CTA_ > 22 showed higher rate of dyslipidemia.

**Figure 2:**
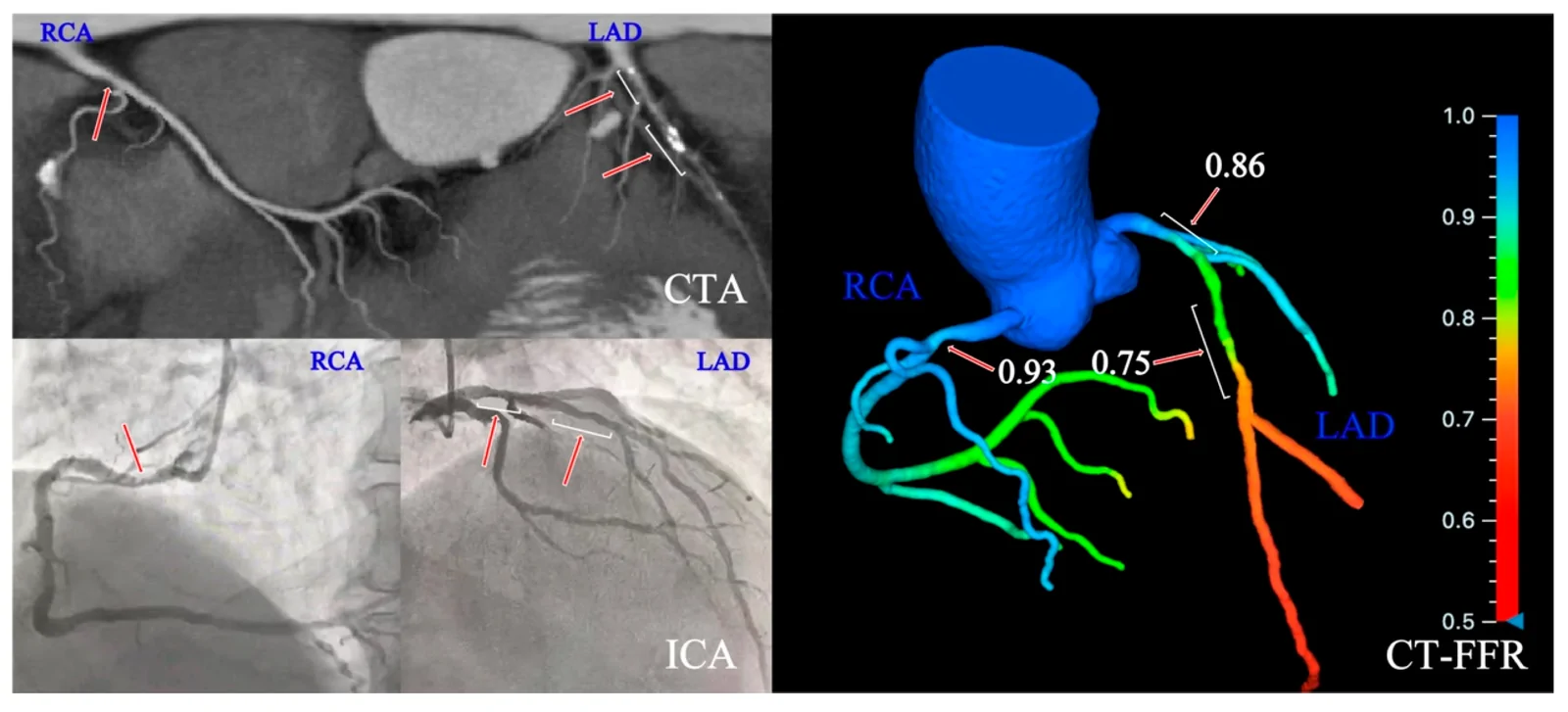
Representative case illustrating the calculation of SS_CTA_, SS_ICA_, and FSS_CTA_ in patient with unstable angina underwent coronary computed tomographic angiography 17 days before invasive coronary angiography. The SYNTAX scores are as follows: **SS_CTA_: 24** (1) LAD proximal to mid-segment lesion with 80% stenosis, corresponding to vessel 6/7, weighted 3.5/2.5 points: (3.5 + 2.5)× 2 = 12 points; type D bifurcation lesion: 2 points. (2) 95–99% stenosis of the first diagonal branch opening (<1.5 mm): no score. (3) LAD mid-segment stenosis, up to 80% stenosis, corresponding to vessel 7, weighted 2.5 points: 2.5 × 2 = 5 points; length > 20 mm: 1 point; severe calcification: 2 points; (4) RCA proximal lesion, most severe 80% stenosis, corresponding to vessel 1, weighted 1 point, should be multiplied by 2 points: 1 × 2 = 2 points. **SS_ICA_: 26** (1) LAD proximal to mid-segment lesion with 75% stenosis, corresponding to vessel 6/7, weighted 3.5/2.5 points: (3.5 + 2.5) × 2 = 12 points; LAD developed 60–70% stenosis at the first septal branch (proximal segment), type D bifurcation lesion: 2 points. (2) 95–99% stenosis at the opening of the first diagonal branch: 1 × 2 = 2 points. (3) LAD mid-segment lesion with 75% stenosis, corresponding to vessel 7, weighted 2.5 points: 2.5 × 2 = 5 points; length > 20 mm: 1 point; severe calcification: 2 points. (4) RCA proximal lesion, most severe 70% stenosis, corresponding to vessel 1, weighted 1 point, should be multiplied by 2 points: 1 × 2 = 2 points. **FSS_CTA_: 8** (The CT-FFR value in the figure is the minimum CT-FFR value within the lesion segment) (1) LAD proximal to mid-segment lesion with 80% stenosis, CT-FFR = 0.86 (>0.8): no score. (2) 95–99% stenosis of the first diagonal branch opening (<1.5 mm): no score (3) LAD mid-segment stenosis, up to 80% stenosis, corresponding to vessel 7, weighted 2.5 points: 2.5 × 2 = 5 points; length > 20 mm: 1 point; severe calcification: 2 points. (4) RCA proximal lesion, most severe 80% stenosis, CT-FFR = 0.93 (>0.8): no score. Abbreviations: CT-FFR, coronary computed tomographic angiography-derived fractional flow reserve; LAD, left anterior descending; RCA, right coronary artery; other abbreviations as in Figure 1.

All patients were divided into two groups based on SS_ICA_, with 697 patients (79.6%) in the low-risk group (≤22) and 179 patients (20.4%) in the intermediate-to-high-risk group (>22) (Table 1). Based on SS_CTA_, 677 patients (77.3%) were divided into the low-risk group and 199 patients (22.7%) into the intermediate-to-high-risk group (Table 2). Based on FSS_CTA_, 743 patients (84.8%) were divided into the low-risk group and 133 patients (15.2%) into the intermediate-to-high-risk group (Table 3). Using the SS_CTA_-based risk rating as a control, SS_ICA_ adjusted 5.6% (49/876) of patients from the intermediate-to-high-risk group to the low-risk group and 3.3% (29/876) from the low-risk group to the intermediate-to-high-risk group. Based on the risk regrouping of FSS_CTA_, 10.4% of patients (91/876) were reclassified to the low-risk group, and 2.9% of patients (25/876) were reclassified to the intermediate-to-high-risk group. A typical case with a downgraded risk classification based on FSS_CTA_ regrouping is shown in Figure 2.

### 3.2. Predictive Role of Three SYNTAX Scores in MACEs

MACEs occurred in 16.6% (145/876) of patients at a median follow-up period of 19.4 months, including four all-cause deaths, 11 non-fatal myocardial infarctions, nine non-fatal strokes, and 121 unplanned revascularizations. Based on SS_ICA_, the proportion of patients with MACEs was significantly higher in the intermediate-to-high-risk group than in the low-risk group (30.2% vs. 13.1%, *p* < 0.001), with a higher proportion of all-cause deaths (1.7% vs. 0.1%, *p* = 0.037), unplanned revascularization (25.1% vs. 10.9%, *p* < 0.001), and no statistical difference between the two groups in terms of non-fatal myocardial infarction and non-fatal stroke (*p* > 0.05) (Table 1). Based on SS_CTA_, the proportion of MACEs was significantly higher in the intermediate-to-high-risk group than in the low-risk group (22.1% vs. 14.9%, *p* = 0.022), while there was no significant difference in each endpoint between the two groups (all *p* > 0.05) (Table 2). Based on FSS_CTA_, the proportion of MACEs in the intermediate-to-high-risk group was significantly higher than that in the low-risk group (36.1% vs. 13.1%, *p* < 0.001), with the proportion of all-cause death (2.3% vs. 0.1%, *p* = 0.008), non-fatal infarction (3.8% vs. 0.8%, *p* = 0.017), and unplanned revascularization (28.6% vs. 11.2%, *p* < 0.001) being higher than that in the low-risk group (Table 3).

**Figure 3:**
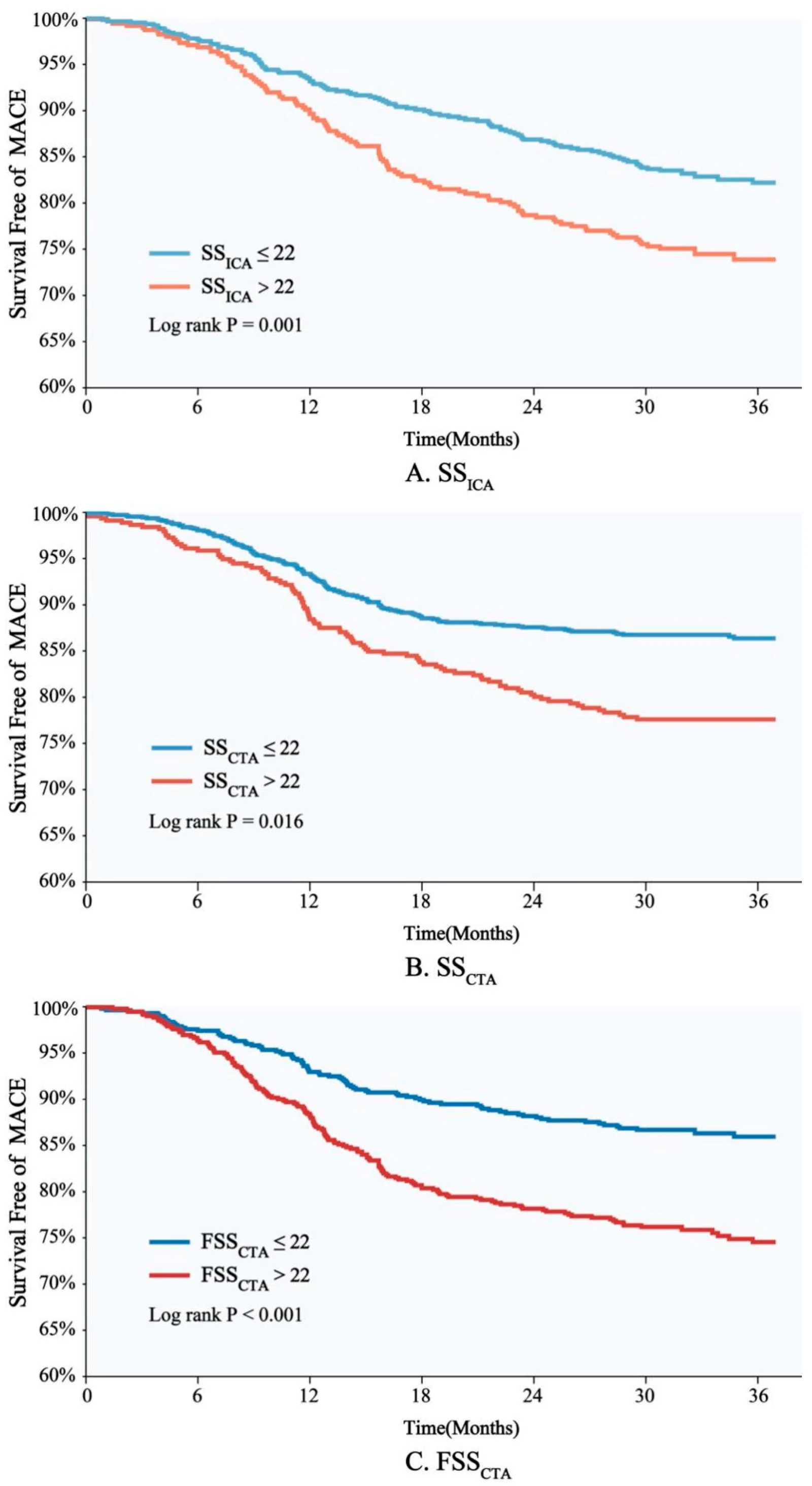
Kaplan–Meier survival curves for MACEs in patients stratified as low-risk or intermediate-to-high-risk according to SS_ICA_ (**A**), SS_CTA_ (**B**), FSS_CTA_ (**C**). Abbreviations: all abbreviations as in Figure 1.

The Kaplan–Meier analysis suggested that patients with SS_ICA_, SS_CTA_, and FSS_CTA_ > 22 had a significantly higher risk of MACEs than those ≤22 (Figure 3). In the univariate analysis of COX regression, all three SS indices were predictors of MACEs, whether as continuous or categorical variables (all *p* < 0.05) (Table 4). In multivariate COX regression analysis, both the FSS_CTA_ (HR 1.082, 95% CI: 1.038–1.128, *p* < 0.001 for continuous variables; HR 1.865, 95% CI: 1.138–2.305, *p* < 0.001 for categorical variables) and SS_ICA_ (HR 1.045, 95% CI: 1.012–1.079, *p* = 0.007 for continuous variables; HR 1.520, 95% CI: 1.128–2.048, *p* = 0.006 for categorical variables) were independent risk predictors of MACEs, while the SS_CTA_ was an independent predictor of MACEs only when considered as a categorical variable (HR 1.318, 95% CI: 1.042–1.667, *p* = 0.021) (Table 4).

In a secondary postprocedural model incorporating complete revascularization and discharge medications, FSS_CTA_ remained an independent predictor of MACEs (HR 1.058, 95% CI 1.015–1.103, *p* = 0.008), while SS_ICA_ (HR 1.030, *p* = 0.065) and SS_CTA_ (HR 1.025, *p* = 0.260) were not significant, consistent with the primary analysis (Supplementary Table S2).

In a prespecified sensitivity analysis restricted to a hard MACE composite (all-cause death, non-fatal MI, and non-fatal ischemic stroke; *n* = 24 events), FSS_CTA_ >22 remained associated with a higher risk of hard MACE (univariable HR 2.803, 95% CI 1.066–7.373, *p* = 0.037; multivariable HR 2.501, 95% CI 0.999–6.254, *p* = 0.050), consistent in direction with the primary analysis. However, given the small number of hard events, these findings should be regarded as exploratory (Supplementary Table S1).

In a subgroup analysis restricted to patients with multivessel disease, FSS_CTA_ remained an independent predictor of MACEs (adjusted HR 1.070, 95% CI 1.014–1.129, *p* = 0.014) (Supplementary Table S4).

Compared with the baseline model (C-statistic 0.581), the addition of each SYNTAX score significantly improved discrimination: C-statistics were 0.699 for SS_ICA_ + BM, 0.694 for SS_CTA_ + BM, and 0.720 for FSS_CTA_ + BM (all *p* < 0.001). Significant improvements in reclassification were also observed for all three models (NRI and IDI, all *p* < 0.05) (Table 5—discrimination ability). For discriminant comparison of MACE risk prediction, FSS_CTA_ + BM outperformed both SS_ICA_ + BM (difference in C-statistic 0.021, *p* = 0.006) and SS_CTA_ + BM (difference 0.026, *p* = 0.002), while SS_ICA_ + BM and SS_CTA_ + BM did not differ significantly (difference 0.005, *p* = 0.100) (Table 5—comparison). Internal validation confirmed the robustness of these findings (Supplementary Table S3).

## 4. Discussion

The Graphical Abstract summarizes the study design, risk stratification, and main prognostic findings. This study investigated the indices of FSS_CTA_, SS_ICA_, and SS_CTA_ to predict MACEs and risk classification in patients undergoing PCI. The FSS_CTA_-based functional SS reclassified the risk rating of patients. FSS_CTA_ and SS_ICA_ were independent predictors of MACEs as both continuous and categorical variables, while SS_CTA_ was an independent predictor of MACEs only as a categorical variable. FSS_CTA_ showed better discriminative performance than SS_CTA_ and SS_ICA_ for MACE risk prediction. In our cohort, ICA- or PCI-related complications occurred in 10 of 876 patients (1.1%), most commonly access-site bleeding or hematoma (0.8%), with no procedure-related deaths. These findings highlight the potential value of non-invasive tools such as CT-FFR for risk stratification, although all patients in this cohort ultimately underwent ICA and PCI. Whether CT-FFR-guided strategies can reduce the need for ICA requires prospective evaluation.

The SYNTAX score is a visual coronary score with an acceptable corelab reproducibility and is useful for a wide range of patients with complex coronary disease [3,14]. It has been widely accepted as a tool to assist in the selection of the optimal revascularization strategy, namely PCI or CABG, and as an independent predictor of poor prognosis in patients with multivessel CAD [15,16]. Additionally, previous studies have suggested that vessels with FFR > 0.8 could be deferred safely for revascularization even for stenosis > 70% [17,18,19]. Therefore, recalculating SS by only incorporating ischemia-producing lesions as determined by FFR may better discriminate risk for adverse events in patients with multivessel CAD undergoing PCI [4,20]. However, to date, there is little published research on the value of FSS_CTA_ for prognosis in patients with complicated CAD.

The addition of functional evaluation allowed the FSS_CTA_ to change the risk classification of the SS based on anatomical assessment. In this study, using the SS_CTA_ risk rating as a control, a total of 13.2% of patients changed in their risk classification based on the FSS_CTA_, with 10.4% of patients reclassified as low-risk and 2.9% of patients reclassified as intermediate-to-high-risk. The SYNTAX II study [10] reported similar results between FSS_CTA_ and FSS_ICA_ among patients with multivessel CAD (21.6 ± 7.8 vs. 21.2 ± 8.8, *p* = 0.589). In other words, calculation of the non-invasive FSS yielded accuracy similar to that obtained with invasive pressure-wire assessment. Furthermore, with the inclusion of the functional component FFR, the agreement between SS_ICA_ and SS_CTA_ for risk classification (low ≤ 22, intermediate 23–32, high ≥ 33) improved from slight to fair agreement [10]. A retrospective single-center study among patients with three-vessel CAD observed that 22.9% of patients were adjusted from the intermediate-to-high-risk group to the low-risk group (FSS_CTA_ ≤ 22) after determining FSS_CTA_, and 52 patients initially referred for CABG based on SS_CTA_ would have been changed to PCI [21].

FSS_CTA_ is a promising prognostic factor in CAD patients. A recent prediction model combining CT-FFR and the SYNTAX score also demonstrated improved risk stratification for MACEs in complex CAD [22]. In the present study, either as a continuous or categorical variable, FSS_CTA_ was an independent predictor of MACEs in patients undergoing PCI with significantly superior performance to SS_CTA_ and SS_ICA_ as estimated by the C-index, NRI, IDI. It is important to note that the primary composite endpoint was predominantly driven by unplanned repeat revascularization. In a sensitivity analysis restricted to hard MACE (death/MI/stroke), FSS_CTA_ > 22 remained associated with a higher risk of hard MACE (multivariable HR 2.501, *p* = 0.051), although the analysis was underpowered because of the small number of events. Neither SS_ICA_ nor SS_CTA_ was significantly associated with hard MACE, consistent with the primary finding that FSS_CTA_ provides better discriminatory value. These findings should be interpreted with caution (Supplementary Table S1). Recent studies have further confirmed the prognostic value of CT-FFR in patients with CAD [19]. Similarly, in a retrospective single-center study [21], the diagnostic efficacy of FSS_CTA_ in predicting MACEs was superior to that of SS_CTA_ (area under the curve: 0.81 vs. 0.75, *p* = 0.01) and SS_ICA_ (area under the curve: 0.81 vs. 0.75, *p* < 0.001) in patients with de novo three-vessel disease. In contrast to the above study, which was restricted to patients with de novo three-vessel disease at a different institution, the present study included consecutive CAD patients undergoing PCI regardless of the number of diseased vessels. In the SYNTAX III REVOLUTION study [23], anatomical SS was reduced by 2.9 points (95% CI 1.9–3.9) after incorporating the non-invasive functional assessment factor CT-FFR, and the proportion of three-vessel CAD patients was reduced by 13.5% (92.3% to 78.8%) after solely including ischemia-causing lesion. The SYNTAX Score III refers to the FSS_CTA_, which combines the SYNTAX II score and CT-FFR, correcting for the overestimation of the predicted 4-year mortality for PCI (FSS_CTA_ vs. SS_ICA_: 16.0% ± 15.8% vs. 14.9% ± 14.7%), with no significant difference for CABG at 4 years [23]. The FSS only calculates ischemic-specific lesions, thereby avoiding the overestimation of lesion complexity evaluated by the SS and more accurately predicting clinical prognosis.

Although the C-statistic was statistically significantly higher with FSS_CTA_ than with SS_CTA_ and SS_ICA_, the absolute improvement was small. Therefore, these findings should be interpreted as demonstrating statistical superiority in discrimination rather than established clinical incremental value. Future studies using decision curve analysis and cost-effectiveness analysis are needed to determine whether FSS_CTA_ improves clinical decision-making and patient outcomes.

Previous studies have validated the potential feasibility of guiding treatment decisions and planning for CAD patients based solely on CT-FFR, especially in complicated CAD patients with three-vessel or left main disease [21,23,24]. The TARGET randomized trial further showed that on-site CT-FFR-guided management reduced the proportion of patients undergoing invasive coronary angiography without obstructive disease, although it did not reduce major adverse cardiovascular events at 1 year [25]. Combining CT-FFR with the SYNTAX II score, FSS_CTA_ changed the therapeutic regimen in 7% (14/196) of patients with left main or three-vessel lesions, including 13 patients who were converted from CABG to PCI [23]. The FASTTRACK CABG study (NCT04142021) [26], an ongoing multicenter prospective study, aims to assess the feasibility and safety of cCTA and CT-FFR as an alternative to ICA as a surgical guide for CABG planning and implementation in patients with three-vessel disease with or without left main lesion. The results of this study may further support the value of CT-FFR in the decision-making of revascularization strategies. In addition, CT-FFR may further improve the diagnostic performance of cCTA during transcatheter aortic valve replacement (TAVR) evaluation, which may potentially reduce the requirement for ICA in the challenging elderly group of patients considered for TAVR [27].

There are several limitations to this study. First, this was a retrospective cohort study with consecutive enrollment; no prospective sample size calculation was performed, and the sample size was determined by the number of eligible patients available during the study period. Second, because patients were consecutively enrolled over a 5-year period, follow-up duration varied (12.5–25.6 months), with later enrollees having shorter follow-up. Third, alcohol consumption was not systematically recorded and could not be included as a baseline risk factor. Fourth, the primary composite endpoint was predominantly driven by unplanned repeat revascularization; in the hard MACE sensitivity analysis, adjustment was limited to age and sex because of the small number of hard events (*n* = 24), and residual confounding cannot be excluded. Fifth, due to clinical practice constraints of invasive FFR in terms of complexity and cost, this study cannot provide evidence of ICA-based FSS (FSS_ICA_), but previous studies have validated the consistency of the FSS_ICA_ and FSS_CTA_ results [10]. Sixth, all patients in this cohort study were treated with PCI; therefore, the impact of the three SS indices (FSS_CTA_, SS_CTA_, SS_ICA_) on the revascularization strategy and the impact of the revascularization strategy on clinical prognosis could not be evaluated. Seventh, external validation in an independent cohort is necessary before clinical implementation. Eighth, the final cohort represents patients in whom AI-based CT-FFR could be successfully analyzed. Exclusions were due to incomplete or inadequate-quality cCTA images rather than clinical selection. Patients with technically challenging CT studies may be underrepresented, which may limit the real-world applicability of our findings.

## 5. Conclusions

FSS_CTA_, SS_CTA_, and SS_ICA_ were all independent predictors of MACEs in CAD patients undergoing PCI, with FSS_CTA_ showing the best discriminatory value. These findings support the potential of FSS_CTA_ for risk stratification in this population.

## Figures and Tables

**Table 1:** Baseline characteristics of study subjects by SS_ICA_.

Variable	SS_ICA_ ≤ 22 (*n* = 697)	SS_ICA_ > 22 (*n* = 179)	*p* Value
MACEs—*n* (%)	91 (13.1)	54 (30.2)	<0.001
All-cause death—*n* (%)	1 (0.1)	3 (1.7)	0.037
Non-fatal MI—*n* (%)	7 (1.0)	4 (2.2)	0.346
Non-fatal stroke—*n* (%)	7 (1.0)	2 (1.1)	1.000
Unplanned revascularization—*n* (%)	76 (10.9)	45 (25.1)	<0.001
Demographics			
Age—years	62.3 ± 9.2	62.1 ± 9.8	0.805
Male—*n* (%)	491 (70.4)	149 (83.2)	0.001
BMI—kg/m^2^	27.2 ± 4.4	27.4 ± 3.4	0.511
Risk Factors			
Current smokers—*n* (%)	207 (29.7)	51 (28.5)	0.823
Hypertension—*n* (%)	392 (56.2)	102 (57.0)	0.925
Dyslipidemia—*n* (%)	503 (72.2)	139 (77.7)	0.166
Diabetes—*n* (%)	244 (35.0)	69 (38.5)	0.427
Past MI—*n* (%)	31 (4.4)	9 (5.0)	0.896
CKD —*n* (%)	20 (2.9)	8 (4.5)	0.397
Lesion Measurements			
GRACE risk score	106 ± 31	110 ± 29	0.105
Complete revascularization—*n* (%)	422 (60.5)	92 (51.4)	0.033
Laboratory Measurements			
hs-CRP—mg/L	1.6 (0.8–3.7)	1.6 (0.7–3.9)	0.433
TC—mmol/L	4.15 ± 1.02	4.19 ± 1.14	0.670
TG—mmol/L	1.48 (0.96–2.09)	1.49 (1.03–2.24)	0.104
LDL-C—mmol/L	2.44 ± 0.89	2.47 ± 0.88	0.412
HDL-C—mmol/L	1.13 ± 0.25	1.14 ± 0.28	0.685
FPG—mmol/L	5.69 (5.20–7.01)	6.08 (5.33–7.09)	0.065
Glycosylated hemoglobin—%	6.0 (5.4–7.0)	6.2 (5.7–7.3)	0.082
Creatinine—μmol/L	70.9 ± 13.3	69.8 ± 10.4	0.159
Medications			
Aspirin—*n* (%)	693 (99.4)	177 (98.9)	0.781
P_2_Y_12_ inhibitors—*n* (%)	697 (100.0)	179 (100.0)	N/A
Statins—*n* (%)	697 (100.0)	179 (100.0)	N/A
ACEI/ARBs—*n* (%)	401 (57.5)	105 (58.7)	0.851
β-blockers—*n* (%)	604 (86.7)	176 (98.3)	<0.001
Oral antidiabetic agents—*n* (%)	219 (31.4)	51 (28.5)	0.505
Insulin—*n* (%)	57 (8.2)	16 (8.9)	0.860

Abbreviations: MACEs, major adverse cardiovascular events; MI, myocardial infarction; BMI, body mass index; CKD, chronic kidney disease; GRACE, Global Registry of Acute Coronary Event; hs-CRP, high-sensitivity C-reactive protein; TC, total cholesterol; TG, triglycerides; LDL-C, low-density lipoprotein-cholesterol; HDL-C, high-density lipoprotein-cholesterol; FPG, fasting plasma glucose; ACEI, angiotensin-converting enzyme inhibitor; ARB, angiotensin II receptor blocker; N/A, not applicable (the *p* value could not be calculated because both groups showed a 100% utilization rate for the indicated medication).

**Table 2:** Baseline characteristics of study subjects by SS_CTA_.

Variable	SS_CTA_ ≤ 22 (*n* = 677)	SS_CTA_ > 22 (*n* = 199)	*p* Value
MACEs—*n* (%)	101 (14.9)	44 (22.1)	0.022
All-cause death—*n* (%)	1 (0.1)	3 (1.5)	0.057
Non-fatal MI—*n* (%)	8 (1.2)	3 (1.5)	0.999
Non-fatal stroke—*n* (%)	6 (0.9)	3 (1.5)	0.716
Unplanned revascularization—*n* (%)	86 (12.7)	35 (17.6)	0.101
Demographics			
Age—years	61.0 ± 9.4	62.2 ± 8.7	0.094
Male—*n* (%)	487 (71.9)	153 (76.9)	0.196
BMI—kg/m^2^	27.1 ± 4.3	27.4 ± 3.6	0.324
Risk Factors			
Current smokers—*n* (%)	194 (28.7)	64 (32.7)	0.317
Hypertension—*n* (%)	388 (57.3)	106 (53.3)	0.352
Dyslipidemia—*n* (%)	491 (72.5)	151 (75.9)	0.396
Diabetes—*n* (%)	237 (35.0)	76 (38.2)	0.459
Past MI—*n* (%)	29 (4.3)	11 (5.5)	0.585
CKD—*n* (%)	19 (2.8)	9 (4.5)	0.327
Lesion Measurements			
GRACE risk score	106 ± 33	111 ± 27	0.030
Complete revascularization—*n* (%)	413 (61.0)	101 (50.8)	0.012
Laboratory Measurements			
h s-CRP—mg/L	1.5 (0.8–3.6)	1.7 (0.8–4.1)	0.345
TC—mmol/L	4.15 ± 1.01	4.21 ± 1.19	0.519
TG—mmol/L	1.48 (0.93–2.10)	1.49 (1.11–2.27)	0.077
LDL-C—mmol/L	2.45 ± 0.87	2.46 ± 0.90	0.890
HDL-C—mmol/L	1.12 ± 0.22	1.14 ± 0.25	0.309
FPG—mmol/L	5.69 (5.22–6.99)	6.11 (5.42–7.06)	0.058
Glycosylated hemoglobin—%	6.0 (5.6–7.0)	6.2 (5.7–7.4)	0.065
Creatinine—μmol/L	70.9 ± 12.2	69.5 ± 15.2	0.110
Medications			
Aspirin—*n* (%)	674 (99.6)	196 (98.5)	0.266
P_2_Y_12_ inhibitors—*n* (%)	677 (100.0)	199 (100.0)	N/A
Statins—*n* (%)	677 (100.0)	199 (100.0)	N/A
ACEI/ARBs—*n* (%)	390 (57.6)	116 (58.3)	0.928
β-blockers—*n* (%)	593 (87.6)	187 (94.0)	0.016
Oral antidiabetic agents—*n* (%)	209 (30.9)	60 (30.2)	0.915
Insulin—*n* (%)	53 (7.8)	20 (10.1)	0.395

Abbreviations: all abbreviations as in Table 1.

**Table 3:** Baseline characteristics of study subjects by FSS_CTA_.

Variable	FSS_CTA_ ≤ 22 (*n* = 743)	FSS_CTA_ > 22 (*n* = 133)	*p* Value
MACEs—*n* (%)	97 (13.1)	48 (36.1)	<0.001
All-cause death—*n* (%)	1 (0.1)	3 (2.3)	0.008
Non-fatal MI—*n* (%)	6 (0.8)	5 (3.8)	0.017
Non-fatal stroke—*n* (%)	7 (0.9)	2 (1.5)	0.901
Unplanned revascularization—*n* (%)	83 (11.2)	38 (28.6)	<0.001
Demographics			
Age—years	62.2 ± 9.3	63.1 ± 9.8	0.326
Male—*n* (%)	548 (73.8)	92 (69.2)	0.322
BMI—kg/m^2^	27.1 ± 4.3	27.4 ± 3.9	0.421
Risk Factors			
Current smokers—*n* (%)	223 (30.0)	35 (26.3)	0.448
Hypertension—*n* (%)	417 (56.1)	77 (57.9)	0.776
Dyslipidemia—*n* (%)	531 (71.5)	111 (83.5)	0.006
Diabetes—*n* (%)	265 (35.7)	48 (36.1)	1.000
Past MI—*n* (%)	36 (4.8)	4 (3.0)	0.478
CKD—*n* (%)	23 (3.1)	5 (3.8)	0.894
Lesion Measurements			
GRACE risk score	107 ± 31	112 ± 30	0.079
Complete revascularization—*n* (%)	449 (60.4)	65 (48.9)	0.017
Laboratory Measurements			
hs-CRP—mg/L	1.6 (0.7–3.6)	1.7 (0.8–3.9)	0.412
TC—mmol/L	4.14 ± 1.12	4.16 ± 1.25	0.863
TG—mmol/L	1.47 (0.99–3.02)	1.48 (1.13–2.33)	0.115
LDL-C—mmol/L	2.44 ± 1.03	2.46 ± 0.98	0.830
HDL-C—mmol/L	1.13 ± 0.25	1.15 ± 0.22	0.345
FPG—mmol/L	5.69 (5.31–7.01)	5.95 (5.25–7.15)	0.118
Glycosylated hemoglobin—%	6.0 (5.3–6.9)	6.1 (5.4–7.1)	0.318
Creatinine—μmol/L	70.4 ± 10.6	69.7 ± 13.9	0.243
Medications			
Aspirin—*n* (%)	741 (99.7)	129 (97.0)	0.003
P_2_Y_12_ inhibitors—*n* (%)	743 (100.0)	133 (100.0)	N/A
Statins—*n* (%)	743 (100.0)	133 (100.0)	N/A
ACEI/ARBs—*n* (%)	423 (56.9)	83 (62.4)	0.279
β-blockers—*n* (%)	661 (89.0)	119 (89.5)	0.982
Oral antidiabetic agents—*n* (%)	225 (30.3)	45 (33.8)	0.475
Insulin—*n* (%)	57 (7.7)	16 (12.0)	0.132

Abbreviations: all abbreviations as in Table 1.

**Table 4:** Cox proportional hazards analyses of three SYNTAX scores to predict MACEs.

	Univariable Analysis		Multivariable Analysis ^a^	
	HR (95% CI)	*p* Value	HR (95% CI)	*p* Value
Continuous				
SS_ICA_	1.056 (1.037–1.107)	<0.001	1.045 (1.012–1.079)	0.007
SS_CTA_	1.039 (1.022–1.056)	<0.001	1.035 (0.981–1.092)	0.208
FSS_CTA_	1.099 (1.039–1.114)	<0.001	1.082 (1.038–1.128)	<0.001
Categorical				
SS_ICA_ > 22	1.589 (1.088–2.310)	0.016	1.520 (1.128–2.048)	0.006
SS_CTA_ > 22	1.365 (1.051–1.997)	0.034	1.318 (1.042–1.667)	0.021
FSS_CTA_ > 22	1.978 (1.317–2.970)	<0.001	1.865 (1.138–2.305)	<0.001

^a^ Multivariable Cox regression model adjusted for age, sex, BMI, current smoking, hypertension, diabetes, dyslipidemia, CKD, and GRACE score.

**Table 5:** Comparative analysis of the discrimination of the three SYNTAX scores added to the baseline model to predict MACEs.

**Discrimination Ability**	**SS_ICA_ + BM ^a^**		**SS_CTA_ + BM**		**FSS_CTA_ + BM**	
	**95%CI**	***p* Value**	**95%CI**	***p* Value**	**95%CI**	***p* Value**
C-Statistic	0.699 (0.667–0.731)	<0.001	0.694 (0.662–0.726)	<0.001	0.720 (0.689–0.751)	<0.001
NRI	0.100 (0.026–0.166)	0.009	0.103 (0.030–0.178)	0.014	0.164 (0.062–0.234)	<0.001
IDI	0.011 (0.003–0.021)	0.008	0.008 (0.002–0.014)	0.022	0.027 (0.009–0.039)	<0.001
**Comparison**	**FSS_CTA_ + BM vs. SS_ICA_ + BM**		**FSS_CTA_ + BM vs. SS_CTA_ + BM**		**SS_ICA_ + BM vs. SS_CTA_ + BM**	
	**Difference**	***p* Value**	**Difference**	***p* Value**	**Difference**	***p* Value**
C-Statistic	0.021	0.006	0.026	0.002	0.005	0.100
NRI	0.064	0.020	0.061	0.007	−0.003	0.358
IDI	0.016	<0.001	0.019	<0.001	0.003	0.202

^a^ Baseline model (BM) including age, sex, BMI, current smoking, hypertension, diabetes, dyslipidemia, CKD, and GRACE score. NRI: net reclassification index; IDI: integrated discrimination improvement. Other abbreviations as in Table 1. C-statistic of BM (95% CI): 0.581 (0.521–0.641), *p* = 0.008.

## Data Availability

The data underlying this article will be shared on reasonable request to the corresponding author.

## Supplementary Materials

The following supporting information can be downloaded at: https://www.mdpi.com/article/10.3390/jcm15207826/s1. Table S1: Sensitivity analysis using a hard MACE composite (all-cause death, non-fatal MI, and non-fatal ischemic stroke), excluding unplanned repeat revascularization; Table S2: Secondary postprocedural model incorporating complete revascularization and discharge medications; Table S3: Internal validation and calibration of the baseline and score-augmented models; Table S4: Subgroup analysis in patients with multivessel disease.

## Abbreviations

ACEI: angiotensin-converting enzyme inhibitor; AI: artificial intelligence; ARB: angiotensin II receptor blocker; AUC: area under the curve; BMI: body mass index; CABG: coronary artery bypass grafting; CAD: coronary artery disease; cCTA: coronary computed tomographic angiography; CI: confidence interval; CKD: chronic kidney disease; CT: computed tomographic; CT-FFR: coronary computed tomographic angiography-derived fractional flow reserve; EPV: events per variable; FFR: fractional flow reserve; FPG: fasting plasma glucose; FSS: functional SYNTAX score; FSS_CTA_: functional SYNTAX score based on CT-FFR; FSS_ICA_: functional SYNTAX score based on invasive coronary angiography; GRACE: Global Registry of Acute Coronary Events; HDL-C: high-density lipoprotein cholesterol; HR: hazard ratio; hs-CRP: high-sensitivity C-reactive protein; ICA: invasive coronary angiography; ICC: intraclass correlation coefficient; IDI: integrated discrimination improvement; IPCW: inverse probability of censoring weighting; IQR: interquartile range; LDL-C: low-density lipoprotein cholesterol; MACEs: major adverse cardiovascular events; MI: myocardial infarction; NRI: net reclassification improvement; NSTEMI: non-ST-elevation myocardial infarction; PCI: percutaneous coronary intervention; SS: SYNTAX score; SS_CTA_: coronary computed tomographic angiography-based SYNTAX score; SS_ICA_: invasive coronary angiography-based SYNTAX score; SYNTAX: synergy between PCI with taxus and cardiac surgery; TAVR: transcatheter aortic valve replacement; TC: total cholesterol; TG: triglycerides; VIF: variance inflation factor.
