# Supplementary material for: Prognostic Implication of a CT-FFR-Based Functional SYNTAX Score in Coronary Artery Disease Patients Undergoing Percutaneous Coronary Intervention
Source: J Clin Med. 2026 Oct 10;15(20):7826. doi: 10.3390/jcm15207826 (PMC13704955; doi:10.3390/jcm15207826)
Supplement: Supplementary file 1 [file jcm-15-07826-s001.zip › jcm-4589824-supplementary.pdf]

Supplementary Table S1. Sensitivity analysis using a hard MACE composite (all-cause death, non-fatal MI, and non-fatal ischemic stroke), excluding unplanned repeat revascularization.

	**Univariable Analysis**		**Multivariable Analysis***	
	**HR (95% CI)**	**p Value**	**HR (95% CI)**	**p Value**
Continuous				
SS_{ICA}	1.042 (0.991–1.096)	0.108	1.033 (0.981–1.088)	0.219
SS_{CTA}	1.036 (0.980–1.095)	0.214	1.027 (0.974–1.083)	0.322
FSS_{CTA}	1.062 (1.003–1.124)	0.039	1.056 (0.994–1.122)	0.078
Categorical				
SS_{ICA}>22	1.904 (0.809–4.502)	0.141	1.753 (0.725–4.253)	0.214
SS_{CTA}>22	1.654 (0.701–3.906)	0.251	1.509 (0.623–3.656)	0.362
FSS_{CTA}>22	2.803 (1.066–7.373)	0.037	2.501 (0.999–6.254)	0.050

*Adjusted for age and sex.

Hard MACE was defined as all-cause death, non-fatal myocardial infarction, or non-fatal ischemic stroke. A total of 24 hard events occurred. HR, hazard ratio; CI, confidence interval.

Supplementary Table S2. Secondary postprocedural model incorporating complete revascularization and discharge medications.

Score	HR (95% CI)	p Value
SS_{ICA}	1.030 (0.998–1.062)	0.065
SS_{CTA}	1.025 (0.982–1.069)	0.260
FSS_{CTA}	1.058 (1.015–1.103)	0.008

Adjusted for age, sex, BMI, current smoking, hypertension, diabetes, dyslipidemia, CKD, GRACE score, complete revascularization, and discharge medications (β-blockers and insulin).

Supplementary Table S3. Internal validation and calibration of the baseline and score-augmented models.

Model	Optimism-corrected C-statistic	Calibration slope	Brier score
Baseline model	0.598	0.95	0.135
SS_{ICA} + BM	0.681	0.93	0.131
SS_{CTA} + BM	0.678	0.94	0.132
FSS_{CTA} + BM	0.695	0.96	0.128

BM, baseline model. Bootstrap resampling was performed with 1,000 replicates.

Supplementary Table S4. Subgroup analysis in patients with multivessel disease.

Score	HR (95% CI)	p Value
SS_{ICA}	1.038 (0.998–1.080)	0.063
SS_{CTA}	1.030 (0.968–1.096)	0.354
FSS_{CTA}	1.070 (1.014–1.129)	0.014

*Adjusted for age and sex.
